# Bushfires, COVID-19 and Young People’s Climate Action in Australia

**DOI:** 10.1007/s10393-022-01595-7

**Published:** 2022-06-06

**Authors:** Hasini Gunasiri, Yifan Wang, Teresa Capetola, Claire Henderson-Wilson, Rebecca Patrick

**Affiliations:** School of Health and Social Development, Faculty of Health, Deakin University, 221 Burwood Highway, Burwood, VIC 3125 Australia

**Keywords:** bushfires, COVID-19, climate change, young people

## Abstract

Australia’s summer bushfires of 2020–2021 were catastrophic, negatively impacting people, and the natural environment. This climate change-related event exacerbated the influence of the COVID-19 pandemic on public health. Young people are a priority population whose health and livelihoods are significantly impacted by these events. At the same time, young people are active agents for climate action. This exploratory mixed-method study draws on descriptive analyses of survey data (*n* = 46) and thematic analyses of interview data (*n* = 6) which demonstrated that some young people, whilst concerned about existential and real impacts of climate change, use contact with nature to cope and as motivation for taking climate actions.

**Supplementary Information:**

The online version contains supplementary material available at 10.1007/s10393-022-01595-7.

## Introduction

Australia faced at least three public health emergencies in 2020—ongoing drought, the catastrophic Black Summer bushfires and the COVID-19 pandemic. These emergencies coincided with the impacts of record-breaking low rainfall averages, extreme heat and droughts during 2017–2019. Emerging evidence suggests that young people have been significantly affected by these crises (Hawke et al., 2020; Beyond Blue, 2021; Lykins, 2020; Danese and Smith, 2020), which are linked by their origins as ecological determinants of health and increasing human pressures on the natural environment, including climate change (WHO, 2020). Further, young people are significantly impacted by and worried about climate change (Sustainability Victoria, 2020; Fritze et al., 2008) and they appear to be one of the most actively engaged groups in climate action (Bandura and Cherry, 2019).

This short research article reports on exploratory data about the impacts of COVID-19 and the Black Summer bushfires on young people in Australia. The paper provides timely social science insights for ecohealth. Specifically, the paper describes how two public health emergencies influenced young people’s lives, including motivations about and capacity to act on, climate change.

## Methodology

The study used an exploratory mixed-method approach involving young people aged 18–24 years residing in Australia. Participatory research principles guided the study design, and it was co-led by two researchers from this age group. Our goal was to co-create knowledge and promote action for social change by involving young people and community partners in all stages of the study’s planning and implementation (Wallerstein & Duran, 2006). This study was approved by Deakin University's Human Ethics Committee.

Convenience and criterion sampling strategies were used to recruit participants (*n* = 46) to a quantitative cross-sectional online survey hosted on Qualtrics from 13 July to 3 August 2020. The recruitment of participants involved two steps. Firstly, young people engaged in climate action were reached through researchers’ existing climate networks and organisations via e-mail and social media. Secondly, managers of youth and health organisations were invited to advertise for study participants in the general young people population through their networks (e.g. social media post and/or emailed advertisement in a newsletter). A mix of climate action engaged, and general population of, young people was deemed important to minimise bias and generate a cross-section of young people’s perspectives. As the researchers did not have access to organisation or network databases, precise reach and participation rates of the online survey could not be obtained (Creswell & Creswell, 2018). All participants provided informed consent to participate in the study. The survey consisted of 13 questions, including validated and new scales, and was pre-tested by young people and academics (see supplementary data table). The final sample included 21 young people involved in climate change/environmental networks or activities and 25 young people from the general population. The mean age was 21 (SD = 1.92).

Purposeful criterion sampling strategies were used to recruit participants (*n* = 6) engaged in climate action in key-informant interviews. The criteria for sampling included young people who identify as being actively engaged in taking action on climate change. Criterion sampling of young people engaged in climate action provided an important qualitative component to the themes generated in the quantitative data and provided specific insights into motivations for, and capacity to act on, climate change. The qualitative semi-structured interview protocol included questions on motivations for engagement with climate action and the impact of COVID-19 and Black Summer bushfires on young people’s lives. Interviews ranged between 10 and 40 min and were led by a researcher aged 25. They were conducted by phone (*n* = 1), zoom (*n* = 5) and digitally recorded.

Quantitative data analysis was undertaken with Excel and STATA 16.0 with descriptive analysis. Qualitative data analysis was undertaken using thematic analysis techniques combined with inductive and deductive approaches (Roberts et al., 2019). Both data sets were triangulated and interpreted in relation to existing literature and guided by planetary health thinking.

## Results

Whilst study participants had direct experiences of a range of climate-related events in their lifetime, including heat and droughts, this short paper focuses on bushfire themes as the interviews highlighted several Black Summer examples. Further, this was deemed a major and more immediate climate-related event and public health emergency experienced by many young Australians across urban and rural settings.

Thirty-four (74%) survey participants reported they had a direct experience of a climate-related event and 12 (26%) reported “none”. Bushfires (*n* = 19, 41%) were among the top three experiences which were chosen by participants (Table 1). There were two participants who chose *other* and listed “smoke from bushfires”.

**Table 1:** Frequency of the Participants’ Direct Experience with Climate-Related Events.

	Whole group
Heatwaves	30 (65%)
Drought	24 (52%)
Bushfires	19 (41%)
Flood or heavy rainfall events	15 (33%)
None	12 (26%)
Cyclone	2 (4%)
Other: Please list	2 (4%)

Impacts of bushfires on regional areas and the environment and endangered species were identified by the study participants. Interview participants were aware of the impact of the 2020–2021 bushfires on regional areas, and they identified the need to prevent them from happening in the future.

…A lot of kids in regional areas are probably thinking, um, they don’t want that to happen to them. So how can we try and stop that from happening in the future (Interviewee 5- age 20).

Study participants expressed their concerns about the impact of bushfires on the environment and endangered species and how climate change contributes to this. They were able to make links between distal and proximal impacts of climate change and referenced the polar bear, which O’Neil et al. (2008) argues has become a popular climate change media icon. This is reflected through the following quote.

…bushfire was a recent one and how the massive bushfires we had in summer are really impacting endangered species already and bigger through climate change, and you hear about the polar bears and the icecaps and all that kind of stuff (Interviewee 6- age 24).

Participants identified awareness raising about climate change as a positive aspect of bushfires. They saw this as an opportunity to reach a wider audience:

I remember at that time a lot of people were posting or sharing climate change related news stories and facts and all that because of how clearly the bushfires were related to increasing levels of climate change (Interviewee 6- age 24).

Participants felt that bushfires provided evidence about climate change and motivated involvement in climate action.

So that, as bad as it was, that was a good thing for promoting the movement because it was like, “Here’s living evidence, here’s evidence that this is happening.” I noticed, for a fact, a lot more people wanted to get involved afterwards… (Interviewee 3- age 23).

**Figure 1:**
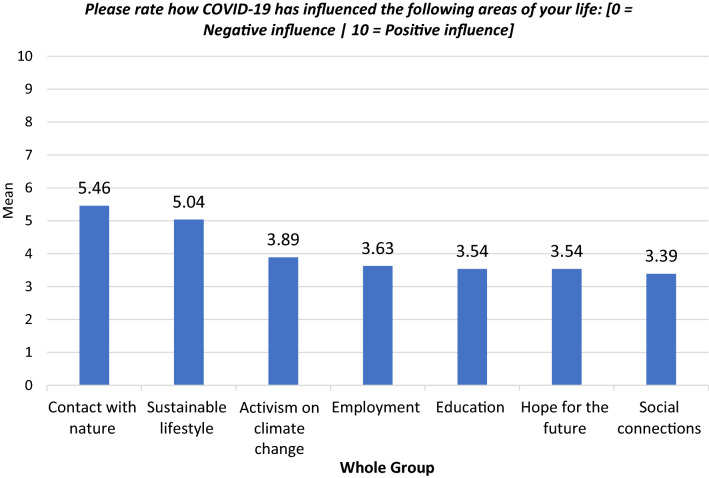
Bar chart of the influence of COVID-19 on different areas of life—whole sample.

Study participants also reported on the influence of COVID-19 on different areas of their lives (Fig. 1).

Several areas of life, including “activism on climate change” (mean:3.89), “employment” (mean:3.63), “education” (mean:3.54), “hope for the future” (mean:3.54) and “social connections” (mean:3.39): [0 = negative influence | 10 = positive influence], were all impacted negatively by COVID-19. Conversely, COVID-19 positively influenced young people’s “contact with nature” (mean:5.46) and “sustainable lifestyle” (mean:5.04).

Interview participants spoke of difficulties in focusing on climate change action due to the pandemic and related impacts. They spoke about the importance of mental health during the pandemic:

I know, for myself, that’s made it very difficult to focus on the issue of climate change because I’ve got to deal with this now, it’s sitting there in the more urgent pile. So, I myself have definitely noticed that I’ve stood back a little bit and taken more time for myself, than I usually would, during the pandemic (Interviewee 2- age 20).

As a result of the pandemic, climate organisations held their meetings online. Interviewees mentioned that they found it difficult to concentrate in the meetings and it decreased their motivation to engage in climate action.

…online meetings for me are a drain of concentration I don’t get the same um, sort of, you know buzz of sort of, “ah we are making a difference” (Interviewee 1- age 23).

Several interviewees expressed their concern that the pandemic was slowing down climate action and that it would have consequences for the future:

It makes me a little bit worried that we’re not doing as much as we could be, in this time, and that this pandemic, once we get through it, we’re going to be two or three years behind where we would have been (Interviewee 2- age 20).

Some interviewees spoke of how COVID-19 exposed climate and social injustices:

The pandemic I think has brought about people seeing climate injustice as such. Or perhaps more rather than climate injustice just injustice in society in general… (Interviewee 5- age 20)*.*

Other interviewees viewed the pandemic as an opportunity to create change in society. It motivated them to continue climate action:

…society is completely you know, been affected, is gonna bring about an opportunity to create some restructuring or to create change (Interviewee 4- age 21).

## Discussion and Conclusion

Even though 2019–2020 Australian bushfires and other climate change-related events were reported to be a concern by the participants, young people in this study were also able to think optimistically about the situation as an opportunity to raise awareness about climate change. This resonates with prior research by Fung and Adams (2017) who found experience of a climate-related event can motivate engagement in, and action on, environmental issues and Kleres and Weetergren’s (2017) study also who found that collective action can help in enabling and sustaining feelings of hope.

This study found that the COVID-19 pandemic affected participant lifestyles and engagement in climate change-related actions. Unsurprisingly, and consistent with other viewpoints on young people’s mental health including Danese and Smith (2020), the pandemic negatively impacted the social determinants of young people’s health including employment, education and social inclusion. Whilst climate activism was negatively impacted, the pandemic appears to have increased motivation and brought climate change issues into clearer focus for young people. Contact with nature and sustainable lifestyles were positively influenced by COVID-19 which is consistent with Pauso et al. (2021). Significantly, contact with nature and sustainable living are both associated with positive environmental behaviours (Martin et al. 2020).

A limitation of this exploratory study is the small sample size. A more representative sample may be used in the future research to further analyse the study’s insights. This study provides novel findings related to the impacts of COVID-19 pandemic and climate change including the 2019–2020 Black Summer bushfires on young people in Australia. These events variously influenced young people’s motivation and capacity to be actively engaged in climate change action. With escalating impacts of climate change in Australia and young people experiencing a disproportionate burden of environmental driven crisis, it is critical that young people’s voices inform ecohealth thinking and action.

## Supplementary Information

Below is the link to the electronic supplementary material.

Supplementary file1 (DOCX 30 kb)
