# Supplementary material for: Bushfires, COVID-19 and Young People’s Climate Action in Australia
Source: Ecohealth. 2022 Jun 6;19(2):149–53. doi: 10.1007/s10393-022-01595-7 (PMC9169437; doi:10.1007/s10393-022-01595-7)
Supplement: Supplementary file 1 — Supplementary file1 (DOCX 30 kb) [file 10393_2022_1595_MOESM1_ESM.docx]

**Appendix – Supplementary Tables for Review**

Survey questions, variables, data analysis and research questions

| **Question (Q) on Survey** | **Variable** | **Data Analysis** | **Research Question** |
| --- | --- | --- | --- |
| Q1 “What is your age?” | Age | Frequency; Mean; SD | RQ1 & RQ2 |
| Q2 “*Thinking about your own life and personal circumstances, how satisfied are you with: [0 = not satisfied \| 10 = completely satisfied]”* | Personal wellbeing | Mean; SD; Median; Range; Skewness; 95%CI; Independent sample t-test | RQ1 & RQ2 |
| Q3 “When you think about climate change, how do you feel?” | Feelings about climate change | Mean; SD; Range; Regression coefficient test separately with Q4, Q6 and Q7. | RQ1 & RQ2 |
| Q4 *“Have you had any direct experience with the following climate-related events? If so, please tick all that apply:”* | Direct experience with climate-related events | Frequency; Percent; Regression coefficient test with Q3 | RQ1 & RQ2 |
| Q5 *“Which of the following do you think are mental health impacts of climate change for young people? Tick all appropriate responses.”* | Opinions about mental health impacts of climate change | Frequency; Percent | RQ1 & RQ2 |
| Q6 *“People can do the following to cope better with climate change. Please rate how often you participate in the following:”* | Use of coping strategies | Mean; SD; Range; Regression coefficient test with Q3 | RQ1 & RQ2 |
| Q7 *“Which of these climate or environmental activities/actions have you been involved in? Please tick all appropriate responses:”* | Participation in climate or environmental activities/actions | Mean; SD; Range; Regression coefficient test with Q3 | RQ1 & RQ2 |
| Q8 *“How does participating in climate change or environmental actions make you feel? Please rate on the following:”* | Feelings associated with participation in different environmental activities | Mean; SD; Range | RQ1 & RQ2 |
| Q9 *“People are talking a lot about climate change in Australia. When you hear about climate change in these areas of your life rate how you feel? [0 = Pessimistic \| 10 = Optimistic]”* | Feelings related to talking about climate changes in different areas of life | Mean; SD; Range | RQ1 & RQ2 |
| Q10 *“Please rate how COVID-19 has influenced the following areas of your life: [0 = Negative influence \| 10 = Positive influence]”* | Influence of COVID-19 on different areas of life | Mean; SD; Range | RQ1 & RQ2 |
| Q11 “Where are you living?” | Location | Frequency; Percent | RQ1 & RQ2 |
| Q12 *“Are you a member of climate /environmental specific organizations?”* | Membership | Frequency; Percent | RQ1 & RQ2 |
| Q13 *“What is your gender?”* | Gender | Frequency; Percent | RQ1 & RQ2 |

Survey questions, variables, data analysis for the whole group

| **Question (Q) on Survey** | **Variable** | **Data Analysis** | | | | |
| --- | --- | --- | --- | --- | --- | --- |
|  |  | Mean | Percentage | Frequency | Range | SD |
| Q1 “What is your age?” | Age | 21 | - | - | 18-24 | 1.92 |
| Q2 “*Thinking about your own life and personal circumstances, how satisfied are you with: [0 = not satisfied \| 10 = completely satisfied]”* | General life satisfaction | 6.72 | - | - | 2-10 | 1.68 |
|  | Living standard | 8.09 | - | - | 0-10 | 2.09 |
|  | Health | 7.52 | - | - | 1-10 | 2.33 |
|  | Achievement | 6.80 | - | - | 1-10 | 1.82 |
|  | Relationships | 6.91 | - | - | 0-10 | 2.22 |
|  | Safety | 7.33 | - | - | 0-10 | 2.18 |
|  | Community connectedness | 6.11 | - | - | 1-10 | 2.27 |
|  | Future security | 4.96 | - | - | 0-10 | 2.48 |
|  | Subjective wellbeing | 6.82 | - | - | 3.43-9.14 | 1.51 |
| Q3 “When you think about climate change, how do you feel?” | Worried | 3.22 | - | - | 1-4 | 1.01 |
|  | Angry | 2.96 | - | - | 1-4 | 0.89 |
|  | Upset | 2.85 | - | - | 1-4 | 0.94 |
|  | Fearful | 2.80 | - | - | 1-4 | 0.83 |
|  | Tense | 2.76 | - | - | 1-4 | 0.92 |
|  | Hopeful | 2.22 | - | - | 1-4 | 0.70 |
|  | Guilty | 2.20 | - | - | 1-4 | 0.65 |
|  | Calm | 1.67 | - | - | 1-4 | 0.87 |
|  | In control | 1.46 | - | - | 1-4 | 0.69 |
|  | Relaxed | 1.41 | - | - | 1-4 | 0.75 |
|  | Content | 1.33 | - | - | 1-4 | 0.67 |
| Q4 *“Have you had any direct experience with the following climate-related events? If so, please tick all that apply:”* | Heatwaves | - | 65% | 30 | - | - |
|  | Drought | - | 52% | 24 | - | - |
|  | Bushfires | - | 41% | 19 | - | - |
|  | Flood or heavy rainfall events | - | 33% | 15 | - | - |
|  | None | - | 26% | 12 | - | - |
|  | Cyclone | - | 4% | 2 | - | - |
|  | Other: Please list | - | 4% | 2 | - | - |
| Q5 *“Which of the following do you think are mental health impacts of climate change for young people? Tick all appropriate responses.”* | Worry about the future | - | 93% | 43 | - | - |
|  | Eco-anxiety | - | 89% | 41 | - | - |
|  | Stress and anxiety | - | 83% | 38 | - | - |
|  | Feelings of despair | - | 61% | 28 | - | - |
|  | Grief and loss | - | 48% | 22 | - | - |
|  | Loss of sense of place | - | 43% | 20 | - | - |
|  | Other: Please list | - | 8% | 4 | - | - |
|  | None | - | 4% | 2 | - | - |
| Q6 *“People can do the following to cope better with climate change. Please rate how often you participate in the following:”* | Nature | 3.46 | - | - | 2-4 | 0.69 |
|  | Change lifestyle | 2.98 | - | - | 1-4 | 0.93 |
|  | Become informed | 2.91 | - | - | 1-4 | 0.76 |
|  | Think optimistically | 2.41 | - | - | 1-4 | 0.80 |
|  | Debate | 2.33 | - | - | 1-4 | 1.14 |
|  | Influence policy | 2.24 | - | - | 1-4 | 1.08 |
|  | Work with others | 2.20 | - | - | 1-4 | 1.05 |
|  | Protests | 2.09 | - | - | 1-4 | 1.15 |
| Q7 *“Which of these climate or environmental activities/actions have you been involved in? Please tick all appropriate responses:”* | Transport | 0.57 | - | - | -1-1 | 0.78 |
|  | Envir protection | 0.30 | - | - | -1-1 | 0.94 |
|  | Water | 0.24 | - | - | -1-1 | 0.92 |
|  | Energy | 0.24 | - | - | -1-1 | 0.92 |
|  | Food | 0.22 | - | - | -1-1 | 0.96 |
|  | Educational | 0.07 | - | - | -1-1 | 0.98 |
|  | Strikes | -0.04 | - | - | -1-1 | 0.99 |
| Q8 *“How does participating in climate change or environmental actions make you feel? Please rate on the following:”* | Pessimistic--Optimistic | 6.25 | - | - | 0-10 | 2.35 |
|  | Stressed--Calm | 5.16 | - | - | 0-10 | 2.12 |
|  | Out of control--In control | 5.43 | - | - | 0-10 | 2.33 |
|  | Fearful--Fearless | 5.09 | - | - | 0-10 | 2.28 |
| Q9 *“People are talking a lot about climate change in Australia. When you hear about climate change in these areas of your life rate how you feel? [0 = Pessimistic \| 10 = Optimistic]”* | Friends | 5.78 | - | - | 0-10 | 2.48 |
|  | School | 5.54 | - | - | 0-10 | 2.45 |
|  | Home | 5.24 | - | - | 0-10 | 2.20 |
|  | Extra-curricular activities | 5.04 | - | - | 0-10 | 2.80 |
|  | Work | 4.73 | - | - | 0-10 | 2.41 |
|  | Social media | 4.67 | - | - | 0-10 | 2.49 |
|  | Politics/gov | 2.96 | - | - | 0-9 | 2.38 |
| Q10 *“Please rate how COVID-19 has influenced the following areas of your life: [0 = Negative influence \| 10 = Positive influence]”* | Contact with nature | 5.46 | - | - | 0-10 | 2.73 |
|  | Sustainable lifestyle | 5.04 | - | - | 0-10 | 2.40 |
|  | Activism on climate change | 3.89 | - | - | 0-10 | 2.36 |
|  | Employment | 3.63 | - | - | 0-10 | 2.89 |
|  | Education | 3.54 | - | - | 0-10 | 2.44 |
|  | Hope for the future | 3.54 | - | - | 0-10 | 2.37 |
|  | Social connections | 3.39 | - | - | 0-10 | 2.64 |
| Q11 “Where are you living?” | Major Urban | 32.0 | - | - | 11-41 | 8.45 |
|  | Outer Urban | 34.5 | - | - | 27-42 | 3.90 |
|  | Regional Town | 36.9 | - | - | 26-42 | 5.43 |
|  | Rural Area | 37.0 | - | - | 37-37 | - |
| Q12 *“Are you a member of climate /environmental specific organizations?”* | Member group | - | 46% | 21 | - | - |
|  | Non-member group | - | 54% | 25 | - | - |
| Q13 *“What is your gender?”* | Female | 34.7 | - | - | 17-42 | 5.82 |
|  | Male | 31.6 | - | - | 11-40 | 8.71 |
|  | Prefer not to say | 26.0 | - | - | 26-26 | - |
